# Broad-Complex Mediates 20-Hydroxyecdysone-Induced Antimicrobial Peptide Expression in the Silkworm *Bombyx mori*

**DOI:** 10.3390/insects17101076

**Published:** 2026-10-17

**Authors:** Pengjie Xian, Tingting Deng, Xiaoqin Li, Yuxin Zhang, Xin Guo, Liang Wang, Xiu He

**Affiliations:** 1 Institute of Basic Medicine, North Sichuan Medical College, Nanchong 637100, China; xianpengjie@126.com (P.X.); 18982597069@163.com (T.D.); lxq_20262026@163.com (X.L.); 18382528392@163.com (Y.Z.); 2 Department of Chemistry, School of Pharmacy and Institute of Pharmacy, North Sichuan Medical College, Nanchong 637100, China

**Keywords:** ecdysteroid, immune, broad-complex, AMP

## Abstract

The steroid hormone 20-hydroxyecdysone (20E) is a key regulator of insect development and has recently emerged as an important modulator of innate immunity. However, the molecular mechanism by which 20E regulates antimicrobial peptide (AMP) expression in lepidopteran insects remains poorly understood. Here, we investigated the role of the 20E signaling pathway in antibacterial immunity in the silkworm *Bombyx mori*. Pretreatment with 20E significantly increased larval survival following infection with both *Escherichia coli* and *Bacillus bombyseptieus* and accelerated bacterial clearance in vivo. Consistently, 20E rapidly induced the expression of multiple AMP genes in the fat body in a dose- and time-dependent manner. Promoter analysis revealed that Broad-Complex (BrC) binding motifs were highly enriched in most AMP promoters. Functional analyses demonstrated that BrC-Z isoforms selectively activated different AMP promoters, with *Leb3* showing the strongest response. Site-directed mutagenesis and electrophoretic mobility shift assays confirmed that BrC-Z directly binds to the *Leb3* promoter to activate transcription. We further identified a previously uncharacterized splice variant, BrC-N, which lacks the zinc-finger DNA-binding domain and cannot activate transcription independently but markedly enhances BrC-Z-dependent *Leb3* activation, likely through BTB/POZ-mediated protein interactions. Together, these findings establish a 20E–BrC–AMP regulatory axis underlying silkworm antibacterial immunity and reveal a previously unknown mechanism by which a noncanonical BrC isoform potentiates hormone-dependent immune gene expression.

## Simple Summary

Silkworms are vulnerable to bacterial infections, but how hormones help them fight these infections remains poorly understood. In this study, we investigated whether 20-hydroxyecdysone, a hormone that controls insect growth and development, also strengthens the natural immune defenses of silkworms. We found that treating silkworm larvae with this hormone before bacterial infection improved their survival and helped them eliminate bacteria more rapidly. The hormone also increased the activity of several genes responsible for producing antimicrobial peptides, small proteins that help defend against harmful microbes. Further experiments revealed that a protein called Broad-Complex plays an important role in activating these immune defense genes. Different forms of this protein regulated different genes, and a newly identified form, although unable to activate genes on its own, significantly enhanced the activity of other forms in promoting the expression of a specific antimicrobial peptide gene. These findings reveal a previously unknown mechanism linking insect development hormones to immune defense and provide new insights into how silkworms protect themselves against bacterial infections. This knowledge may contribute to future strategies for improving silkworm health and disease resistance.

## 1. Introduction

Insects are the most diverse and widely distributed group of animals on Earth. During evolution, they developed an efficient and complex innate immune system to defend against a wide range of pathogens [1]. Unlike vertebrates, insects do not possess antibody-mediated adaptive immunity [2]. Therefore, they rely entirely on innate immunity for pathogen recognition and elimination. The insect innate immune system consists of two major branches: cellular immunity and humoral immunity [3].

Antimicrobial peptides (AMPs) are among the most important effector molecules of insect humoral immunity [4,5]. The first insect AMP, cecropin, was isolated from the pupae of the giant silk moth *Hyalophora cecropia* [6]. Since then, hundreds of AMPs have been identified in insects. Most AMPs are synthesized in the fat body and secreted into the hemolymph. They are small, heat stable, and active against a broad range of pathogens [7,8,9,10,11]. In the silkworm (*Bombyx mori*), at least 35 AMP genes have been identified [12]. Based on sequence similarity, they are classified into seven families: Cecropin, Attacin, Moricin, Gloverin, Lebocin, Enbocin, and Defensin [12]. These peptides are essential for host defense against microbial infection. Their expression is controlled by a precise and complex regulatory network [5,13].

The steroid hormone 20-hydroxyecdysone (20E) is one of the major hormones in insects. It plays essential roles in molting, metamorphosis, reproduction, and behavior [14]. After binding to its nuclear receptor complex EcR/USP, 20E initiates a transcriptional cascade that regulates the expression of many downstream genes, including numerous transcription factors [15]. Increasing evidence has shown that 20E functions not only as a developmental hormone but also as an important regulator of insect immunity [16,17,18,19,20,21,22]. The endogenous level of 20E changes significantly after pathogen infection, suggesting that this hormone actively participates in immune responses. In *Drosophila* and mosquitoes, 20E has been shown to induce the expression of several AMP genes [16,17,23,24]. This regulation occurs through at least two mechanisms. One mechanism depends on the immune deficiency (IMD) pathway. In this pathway, 20E increases the expression of the peptidoglycan recognition protein PGRP-LC, which subsequently activates AMP gene expression. The other mechanism is independent of PGRP-LC. Instead, 20E regulates specific AMP genes through downstream transcription factors [16]. Because 20E regulates both development and immunity, disrupting key components of the 20E signaling pathway, such as EcR or downstream transcription factors, may simultaneously impair insect growth and immune defense. However, in lepidopteran insects, the molecular mechanism by which 20E controls AMP expression through downstream transcription factors remains poorly understood. In particular, the key transcription factors that function as molecular switches have not been systematically identified.

Broad-Complex (BrC) is an important early-response transcription factor in the 20E signaling pathway [25,26,27]. BrC belongs to the C2H2 zinc-finger transcription factor family [28]. Alternative splicing generates several protein isoforms, including Z1, Z2, and Z4. All isoforms share a conserved BTB/POZ domain at the N terminus. This domain mediates protein–protein interactions and dimerization. The C termini contain different zinc-finger domains that recognize and bind specific *cis*-regulatory elements in target gene promoters [29,30,31,32]. BrC is specifically expressed during the pupal stage and is required for larval tissue degeneration and adult imaginal disc differentiation [33,34,35,36,37]. In this study, we used the silkworm as a model to investigate how 20E regulates AMP gene expression through BrC. We focused on the distinct functions of different BrC splice isoforms in controlling AMP promoters. We also characterized a novel splice variant, BrC-N, and examined its potential role in cooperative transcriptional regulation. Our findings provide new insights into the crosstalk between endocrine signaling and innate immunity in insects. They also offer potential molecular targets for the development of new pest control strategies.

## 2. Materials and Methods

### 2.1. Insect Materials, Cell Culture, and Pathogens

The bivoltine silkworm strain *Dazao* (*p50*) was used in this study. Larvae were reared on fresh mulberry leaves at 25.0 °C with 65–80% relative humidity. All experiments were performed using healthy fifth-instar day 3 larvae. Day-3 fifth-instar larvae were chosen because endogenous 20E titers are known to be very low at this developmental stage [38], and ecdysone signaling activity is correspondingly low [39]. Therefore, this stage provides a sensitive baseline for detecting infection-induced changes in hormone signaling. The silkworm ovary-derived cell line *BmNs* was cultured at 27 °C in TC-100 insect medium (PAN-Biotech, Aidenbach, Germany) supplemented with 10% fetal bovine serum (Gibco, Grand Island, NY, USA) under sterile conditions.

Two bacterial strains were used for infection assays: the Gram-negative bacterium *Escherichia coli* and the Gram-positive bacterium *Bacillus bombyseptieus*.

### 2.2. Bacterial Infection and Bacterial Clearance Assays

Bacterial strains were cultured overnight with shaking, washed with sterile physiological saline, and adjusted to the required concentrations before injection. Healthy fifth-instar day 3 silkworm larvae were selected for infection experiments.

For survival assays, larvae were divided into control and infection groups. Before bacterial challenge, each larva was pretreated with 0.5 μg 20E, while control larvae received the same volume of dimethyl sulfoxide (DMSO). Twelve hours later, larvae were injected with *E. coli* (10^4^ CFU per larva) or *Ba. bombyseptieus* (10^3^ CFU per larva). Survival rates were recorded at different time points after infection.

For bacterial clearance assays, *Ba. bombyseptieus* was heat-inactivated at 70 °C and labeled with fluorescein isothiocyanate (FITC) before injection. Prior to bacterial infection, larvae were injected with 20E (0.5 μg per larva). Hemolymph samples were collected at 24 h after infection. FITC-labeled bacteria were observed using a fluorescence microscope.

### 2.3. Fat Body Collection and 20E Treatment

In vitro 20E induction: Fat bodies were dissected from fifth-instar day 3 larvae and cultured in Grace medium in six-well plates. For the treatment groups, different concentrations of 20E were added to the medium at final concentrations of 0.4 μg/mL (high dose) and 0.04 μg/mL (low dose). For the control group, DMSO was added. Because dissected fat bodies are more directly and sensitively stimulated by 20E than in vivo injection, tissues were collected at 1 and 3 h after treatment and immediately frozen in liquid nitrogen for RNA extraction.

In vivo 20E induction: Each fifth-instar day 3 larva was injected with 2 μg 20E. Fat body tissues were collected at 3 and 6 h after injection. Samples were immediately frozen in liquid nitrogen and used for RNA extraction.

### 2.4. RNA Extraction, cDNA Synthesis, and qRT-PCR Analysis

Total RNA was extracted using TransZol reagent (Invitrogen, Carlsbad, CA, USA). After DNase treatment, first-strand cDNA was synthesized using the EasyScript reverse transcription kit (TransGen Biotech, Beijing, China). Quantitative real-time PCR was performed using SYBR Premix Ex Taq II (Takara Bio, Kusatsu, Japan) on a Bio-Rad CFX Manager system. The ribosomal protein gene (RPL3) was used for normalization. All primers are listed in Supplemental Table S1.

### 2.5. Prediction of Transcription Factor Binding Sites and Gene Cloning

The 1500 bp upstream promoter regions of 11 AMP genes were retrieved from the silkworm genome database (Silkbase) [40,41]. Potential transcription factor binding sites (TFBSs) were predicted using the Genomatix Genome Analyzer (Genomatix, Munich, Germany).

Total RNA was extracted from the fat body of *E. coli*-infected silkworms, and 3′RACE was performed to obtain the isoforms of BrC. Briefly, 3′RACE-Ready cDNA was synthesized by reverse transcription using an oligo (dT) primer carrying an adapter sequence by 3′-Full RACE Core Set with PrimeScript™ RTase kit (Takara, Japan). Gene-specific primer (GSPs) and nested inner primer (GSP2) were designed based on the known BrC sequence of *B. mori* in the NCBI database. First-round PCR was carried out with GSP and the adapter primer using cDNA as the template. Then, the first-round product was diluted and subjected to nested PCR with GSP2. The target PCR products were purified and ligated into the pEASY-T1 vector for sequencing verification.

### 2.6. Dual-Luciferase Reporter Assay

The promoter regions of *CecE*, *Glv2*, and *Leb3* were amplified using primers containing Nhe I and Hind III restriction sites and cloned into the pGL3-basic luciferase reporter vector. For construction of the *Leb3* promoter mutant, the predicted core BrC-binding motif located at −231 to −228 bp was mutated from TAAA to GACA using overlap extension PCR. The mutant promoter was then cloned into the pGL3-basic vector to generate Leb3-M/pGL3.

Cell transfection and luciferase activity analysis: *BmNs* cells at the logarithmic growth phase were co-transfected with target expression plasmids and the Renilla luciferase control plasmid RnLc/pIZ at a ratio of 10:1 using X-tremeGENE HP transfection reagent. After 24 h of transfection, cells were treated with different concentrations of 20E (2 μM and 20 μM). Cells were lysed after treatment, and firefly and Renilla luciferase activities were measured using the Promega Dual-Luciferase Reporter Assay System.

### 2.7. Electrophoretic Mobility Shift Assay (EMSA)

BmNs cells were transfected with the BrC-Z4/IEST expression plasmid. After 48 h, nuclear proteins were extracted using the Beyotime Nuclear Protein Extraction Kit. Based on the predicted BrC-Z binding site in the Leb3 promoter, a biotin-labeled DNA probe (5′-TTTCACAGATAGGTAAACAAAATTTACGAC-3′), an unlabeled competitive probe, and a mutant probe were synthesized (Sangon Biotech, Shanghai, China). The probes were annealed and incubated with nuclear proteins in binding buffer containing glycerol, MgCl_2_, poly(dI·dC), and NP-40. The protein–DNA complexes were separated on a 6.5% non-denaturing polyacrylamide gel at 100 V and 4 °C. DNA was transferred onto a positively charged nylon membrane and cross-linked using UV irradiation (120 mJ/cm^2^, 254 nm, 45–60 s). The shifted bands were detected using a chemiluminescence detection kit and visualized by X-ray film exposure.

## 3. Results

### 3.1. 20E Enhances Immune Responses in B. mori

To determine the immuno-regulatory function of 20E in *B. mori* at the organismal level, we performed exogenous hormone treatment followed by bacterial infection assays. We evaluated the effects of 20E pretreatment on host survival and bacterial clearance ability. Two representative pathogens were used for infection experiments: the Gram-negative bacterium *E. coli* and the Gram-positive bacterium *Ba. bombyseptieus*.

**Figure 1:**
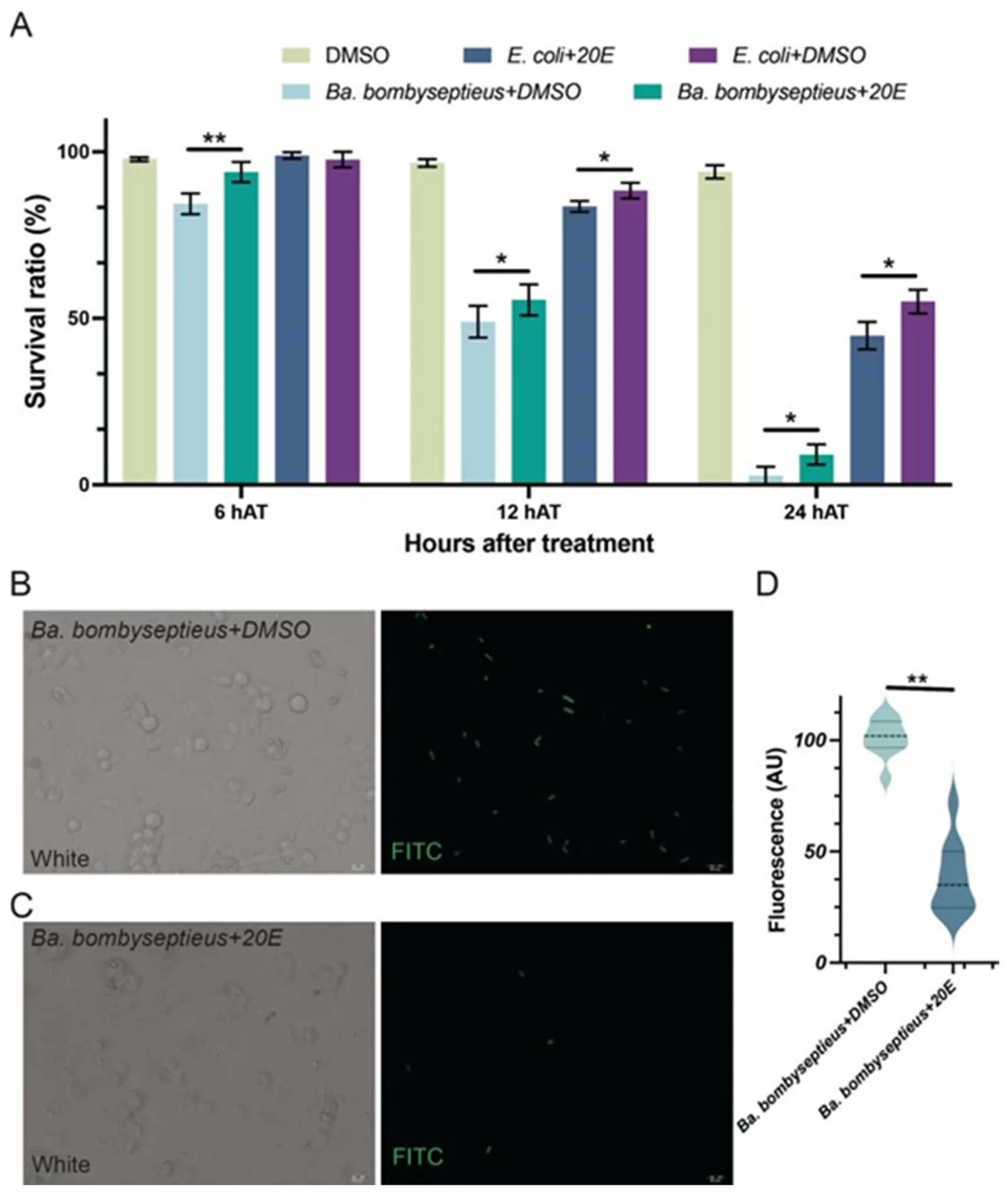
**20E promotes the insect immunity of the silkworm.** (**A**) The survival ratio of silkworm larvae infected with different bacteria after DMSO or 20E treatment. Briefly, 20E (0.5 μg/larva) or DMSO was injected into the hemolymph of the day-3 fifth-instar larvae. Twelve hours later, larvae were injected with *E. coli* (10^4^ cells/larva) or *Ba. bombyseptieus* (10^3^ cells/larva). Sample size (30 larvae per group); the data show mean ± SEM from three independent biological replicates. Significance was calculated using Student’s *t*-test (* *p* < 0.05; ** *p* < 0.01). Clearance of FITC-labeled *Ba. bombyseptieus* at 24 h after DMSO (**B**) or 20E treatment (**C**). (**D**) Quantification of fluorescence intensity of FITC-labeled *Ba. bombyseptieus* after DMSO or 20E treatment. Data show mean ± SEM. Significance was calculated using Student’s *t*-test (** *p* < 0.01).

Compared with the control group, silkworms without 20E pre-treatment showed a rapid decrease in survival rate after bacterial infection. In contrast, silkworms pre-treated with 20E 12 h before infection exhibited significantly higher survival rates at multiple time points (12 and 24 h after infection) (*p* < 0.05) (Figure 1A). These results provide evidence at the individual level that exogenous 20E improves the resistance of silkworms to both Gram-negative and Gram-positive bacterial infections and reduces pathogen-induced mortality.

To further investigate the mechanism underlying 20E-mediated immune protection, we performed an in vivo bacterial clearance assay using FITC-labeled *Ba. bombyseptieus*. At 24 h after infection, the amount of remaining fluorescent bacteria in the hemolymph was significantly lower in the 20E-pretreated group than in the control group (Figure 1B–D). This result indicates that the hormone could activate host immune responses and promote pathogen recognition, encapsulation, and elimination. Together, these results demonstrate that exogenous 20E strongly enhances systemic immune defense in silkworms. One major function of 20E is to improve the ability of immune cells and humoral factors to remove invading pathogens, thereby increasing host survival.

### 3.2. Differential Responses of Antimicrobial Peptide Genes to 20E in the Fat Body

To further understand how 20E promotes immune responses in silkworms, we focused on antimicrobial peptides (AMPs), which are major effector molecules of insect humoral immunity. Because the fat body is the primary organ responsible for AMP synthesis, we examined the transcriptional responses of AMP genes in this tissue after 20E treatment [42]. Fat bodies were isolated from day 3 fifth-instar larvae of the Dazao strain and subjected to both in vitro and in vivo 20E induction assays. Seven AMP genes from four major AMP families, including Cecropin, Gloverin, Lebocin and Moricin, were selected for expression analysis (*CecB1*, *CecD*, *CecE*, *Glv2*, *Glv3*, *Leb3*, and *Mor1*).

**Figure 2:**
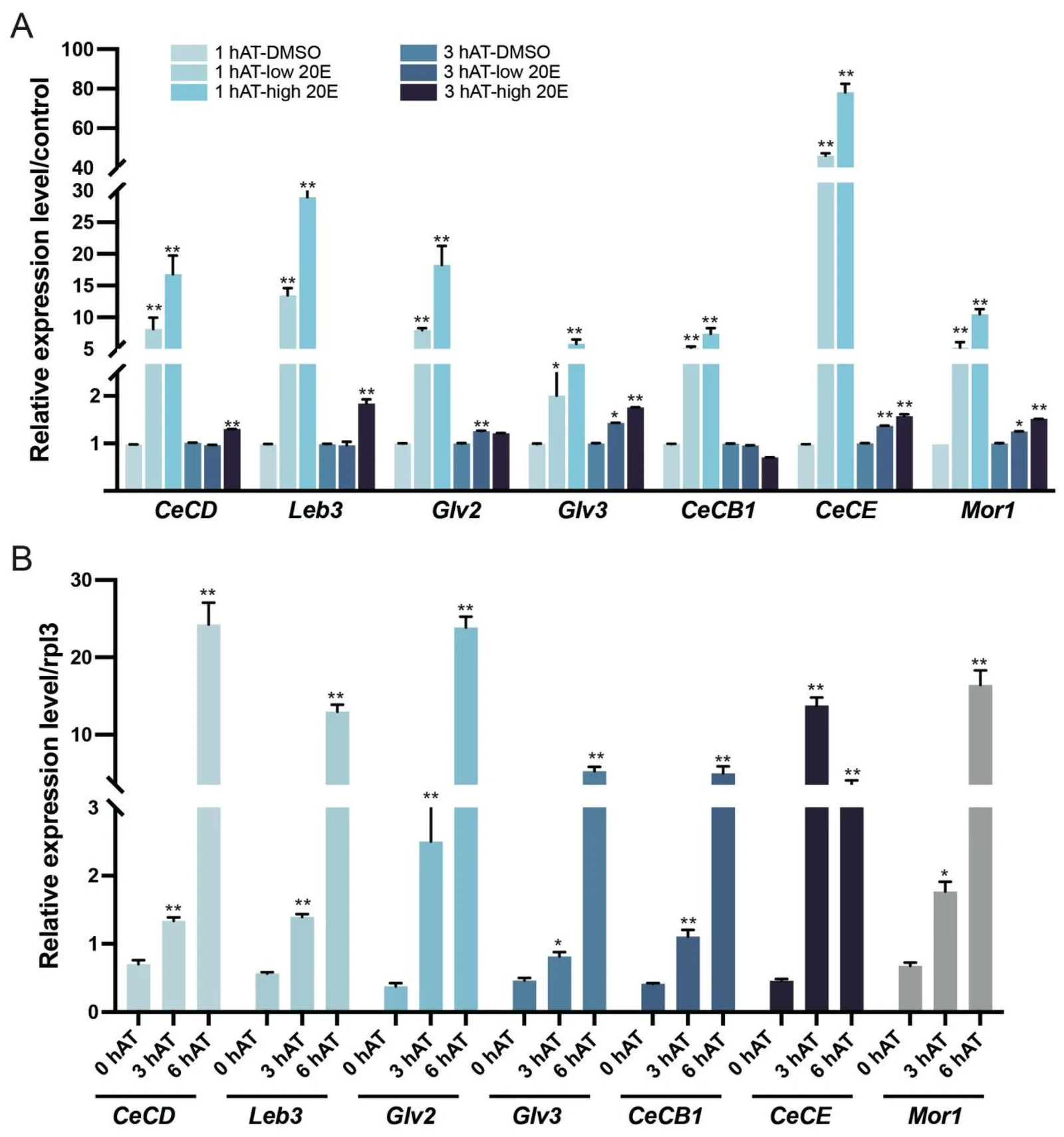
**20E triggers the expression of antimicrobial peptides (AMPs).** (**A**) In vitro induction of AMP expression from isolated silkworm fat body with different dosage of 20E treatment; (**B**) in vivo induction of AMP expression from fat body dissected from silkworm larvae injected with 20E. The data show mean ± SEM from three independent biological replicates. Significance was calculated using Student’s *t*-test (* *p* < 0.05; ** *p* < 0.01).

In the in vitro induction assay, all examined AMP genes were significantly upregulated after 1 h of 20E treatment. Moreover, their expression levels increased in a hormone dose-dependent manner. Interestingly, after 3 h of treatment, the induction effects were generally reduced. However, except for *CecB1*, the expression levels of most AMP genes remained higher than those in the control group (Figure 2A). These results suggest that 20E-induced AMP expression may involve rapid feedback regulation or receptor desensitization, which may prevent excessive immune activation and protect host tissues from immune-related damage.

To further confirm these effects in vivo, we injected 20E into fifth-instar larvae and examined AMP gene expression at different time points (3 and 6 h after injection). Most AMP genes showed a time-dependent increase in expression. Their transcript levels were significantly elevated at 3 h and continued to increase at 6 h, with expression levels rising approximately 5- to 30-fold compared with the initial levels (Figure 2B). In contrast, *CecE* reached its highest expression level at 3 h and then gradually decreased.

Overall, these results demonstrate that 20E positively regulates the expression of multiple AMP genes in the silkworm fat body and contributes to systemic immune activation.

### 3.3. Characterization of Transcription Factor Binding Sites in AMP Promoters

To identify the potential molecular switch responsible for 20E-mediated activation of AMP genes in silkworms, we analyzed the promoter regions of 11 identified AMP genes. The 1.5 kb upstream regions before the ATG start codon were selected for transcription factor binding site (TFBS) prediction using the Genomatix platform. The prediction results showed that, except for *Glv1*, the promoters of the other 10 AMP genes contained multiple binding motifs for 20E early-response factors, including BrC, E74, and FTZ-related elements (Supplemental Table S2). Notably, BrC binding motifs accounted for more than 50% of the predicted transcription factor binding sites in the promoters of *Glv2*, *Glv4*, *CecA2*, *CecB1*, *CecE*, and *Leb3*. This high frequency strongly suggests that the BrC transcription factor family may serve as a central regulator linking 20E signaling to AMP gene activation.

### 3.4. Induction of BrC Variants by Hormones and Pathogens

**Figure 3:**
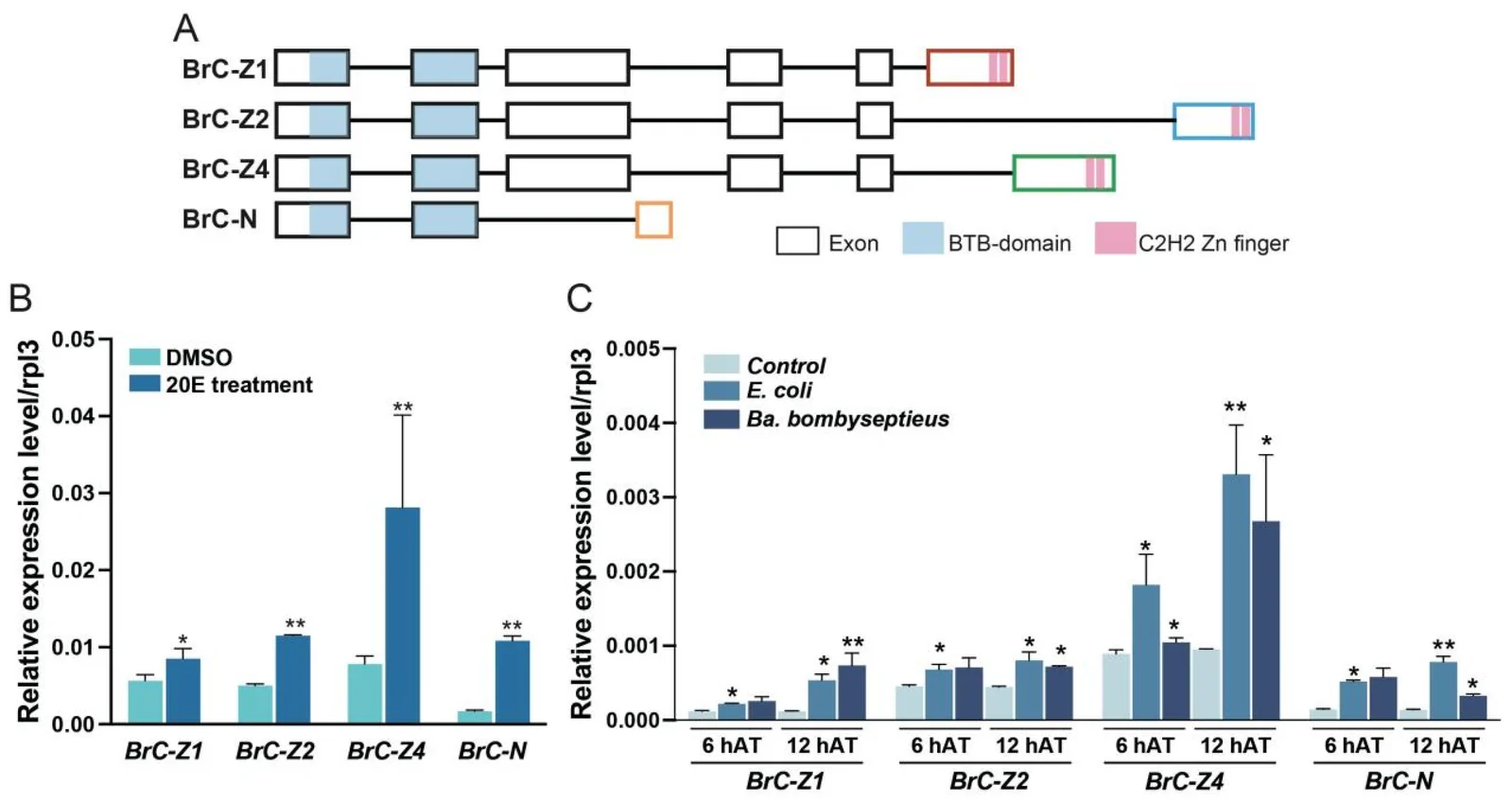
***BrCs* are bacteria and 20E induced transcription factors.** (**A**) Four *BrC* isoforms were identified from the fat body of silkworm larvae infected with *E. coli*. The black box represents the common exons among different *BrC* isoforms; the colorful box represents specific exons for each isoform. The blue filled box represents the location of BTB-domain; the pink filled box represents the location of the C2H2 Zn finger region. (**B**) The expression pattern of four *BrC* isoforms from the fat body of larvae at 6 h after injection with 20E or DMSO. (**C**) The expression pattern of four *BrC* isoforms from the fat body of bacteria-infected larvae. hAT means hours after treatment. The data show mean ± SEM from three independent biological replicates. Significance was calculated using Student’s *t*-test (* *p* < 0.05; ** *p* < 0.01).

To functionally investigate the immune-related role of BrC, we first cloned the annotated BrC homologs from the silkworm genome. Three conserved splice variants, BrC-Z1, BrC-Z2, and BrC-Z4, containing intact zinc finger DNA-binding domains were successfully obtained. Interestingly, we also identified a previously unknown splice variant during the cloning process, which was named BrC-N. Sequence analysis showed that BrC-N was generated through partial exon skipping, resulting in premature termination of the open reading frame (Figure 3A). The predicted protein retained the N-terminal BTB/POZ domain but completely lacked the C-terminal zinc finger domain responsible for DNA recognition (Supplemental Figure S1). Therefore, BrC-N was predicted to lack independent transcriptional activation activity.

Quantitative real-time PCR showed that exogenous 20E treatment significantly induced the expression of all four BrC splice variants in the fat body of silkworm larvae, confirming that all *BrC* isoforms are early response genes of the 20E pathway (Figure 3B). Because previous study has shown that bacterial infection increases endogenous 20E levels in silkworms [22], we next asked whether bacterial infection regulates BrC expression through elevated 20E. Consistent with the previous report, infection with either *E. coli* or *Ba. bombyseptieus* elevated endogenous 20E titers (Supplemental Figure S2), and correspondingly, both bacteria significantly increased the expression of all four BrC isoforms (Figure 3C).

Together, these expression patterns suggest that upon pathogen invasion, silkworms may activate different combinations of the endogenous 20E/BrC signaling axis to regulate specific *BrC* isoforms. This selective regulation may provide a transcriptional basis for the precise activation of AMP genes during immune responses.

### 3.5. The 20E–BrC Signaling Axis Activates AMP Gene Transcription

**Figure 4:**
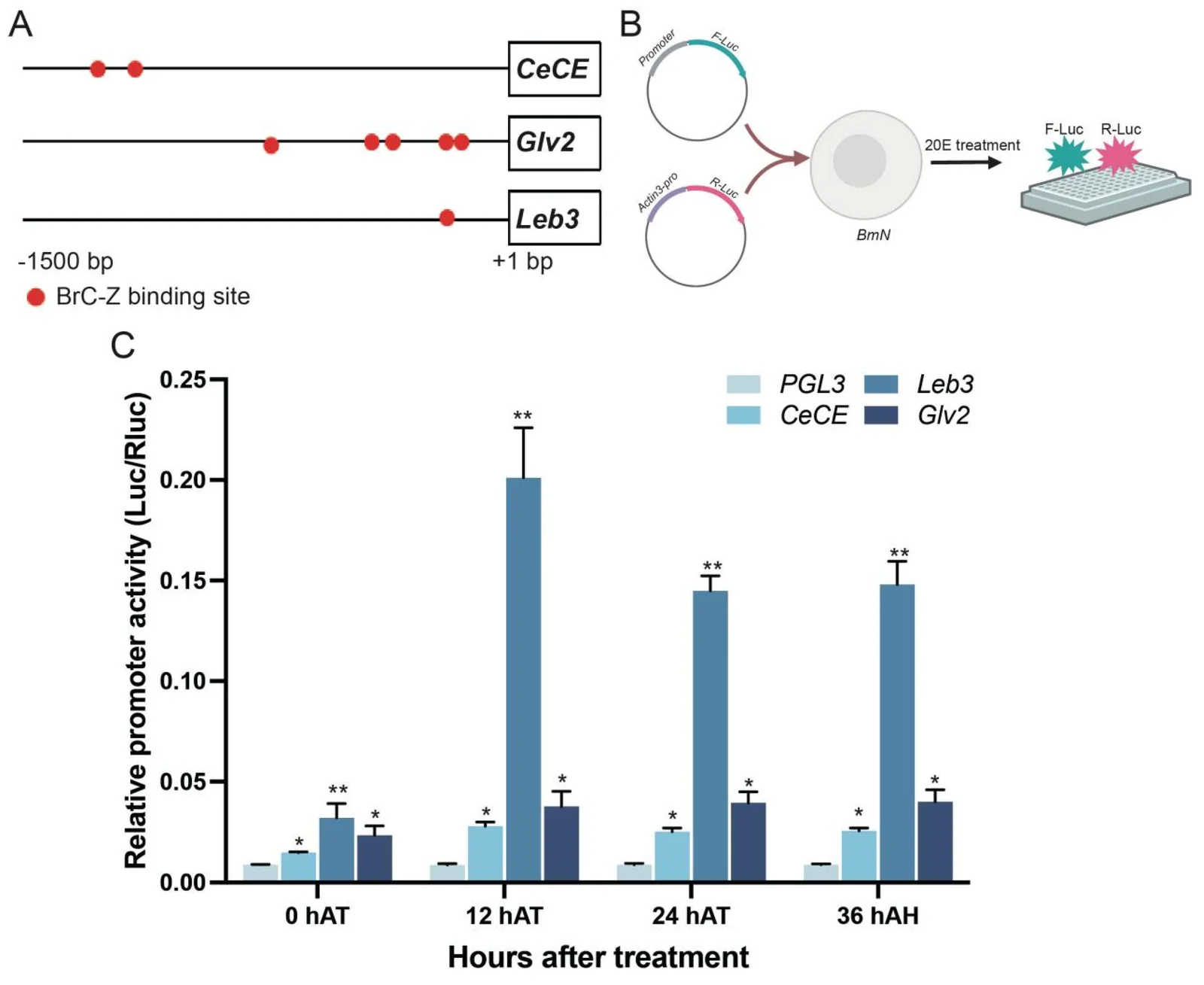
**20E can induce the promoter activity of AMPs.** (**A**) Schematic outline of the regulatory regions with predicted BrC binding sites (red circles) from three AMPs. (**B**) Dual luciferase assay to examine the promoter activity of AMPs after 20E treatment. Plasmids containing regulatory regions of AMPs (Promoter) driving firefly luciferase (F-luc) were co-transfected with a Renilla luciferase (R-luc) reporter driven with *actin3* promoter (actin3-pro) into *BmN* cells. After 24 h of transfection, 20E was added to the medium. The luciferase activity was then measured. (**C**) The relative promoter activity of three AMPs after 20E treatment. hAT means hours after 20E treatment. The data show mean ± SEM from three independent biological replicates. Significance was calculated using Student’s *t*-test (* *p* < 0.05; ** *p* < 0.01). Statistical significance for basal promoter activity was determined by comparing each AMP promoter reporter with the empty pGL3 vector. For time-course analyses, each time point was compared with the corresponding 0 h after treatment (0 h AT) control of the same AMP promoter reporter.

To further clarify the direct regulatory mechanism of the 20E/BrC signaling axis on AMP genes, we selected three representative AMP genes (CecE, Glv2, and Leb3) that showed strong responses to 20E treatment. The 1.5 kb upstream regulatory regions of these genes were cloned into the firefly luciferase reporter vector (Figure 4A). The reporter constructs were then co-transfected with the Renilla luciferase control plasmid into silkworm *BmN* cells. Promoter activity was analyzed using a dual-luciferase reporter assay (Figure 4B).

Under basal conditions without 20E treatment, all three AMP promoter reporters showed significantly higher luciferase activity than the empty vector control. This indicates that the cloned 1.5 kb promoter regions are sufficient to drive basal transcription (Figure 4C). After 20E treatment, the promoter activities of all three AMP genes were significantly increased. Among them, the Leb3 promoter showed the strongest response to 20E stimulation (Figure 4C). These cellular results were consistent with the AMP expression patterns observed in both in vivo and in vitro experiments, confirming that this reporter system is suitable for studying hormone-dependent transcriptional regulation.

**Figure 5:**
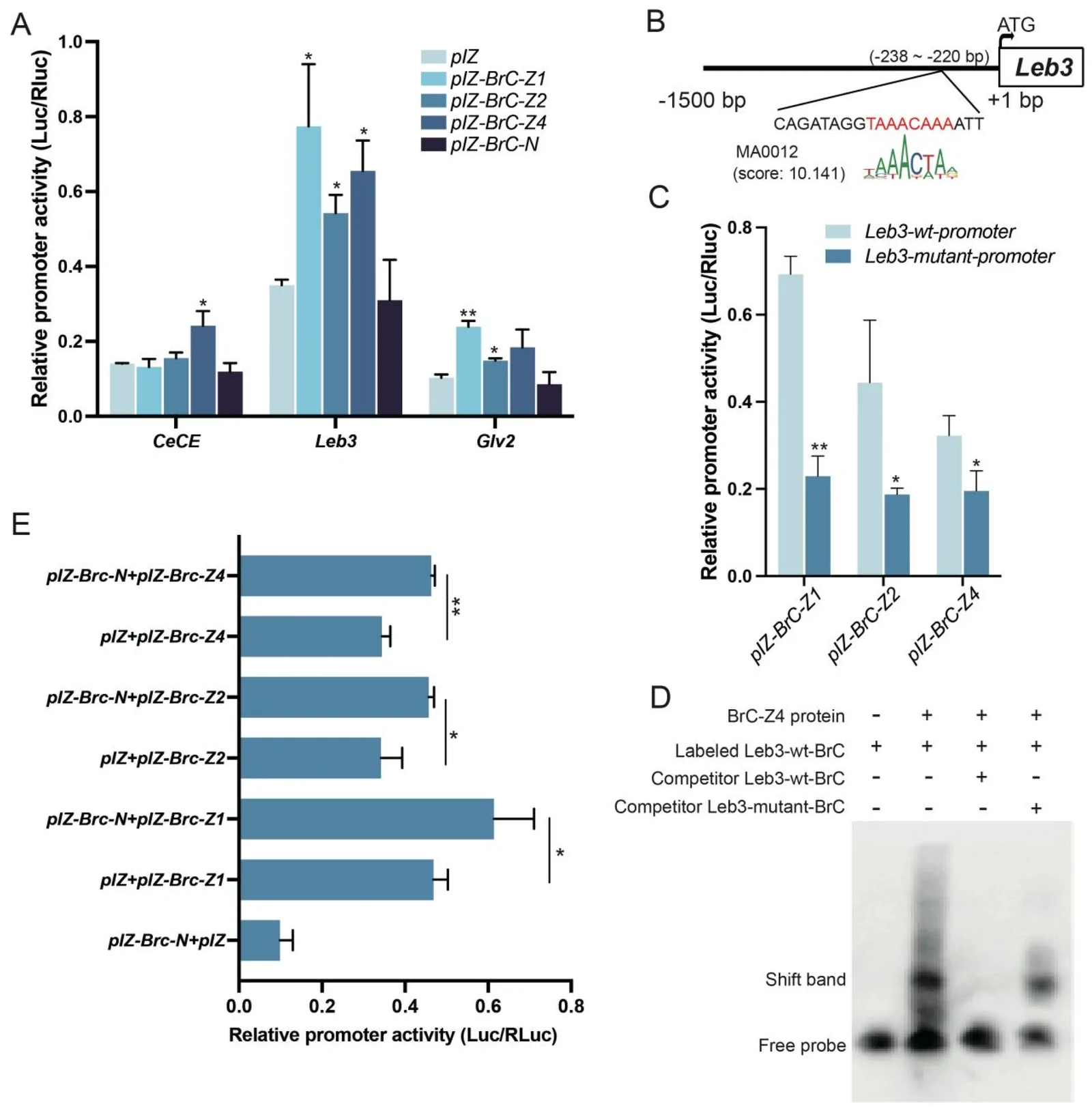
**BrC can bind to the promoter region of Leb3.** (**A**) The relative promoter activity of AMPs after overexpressing four BrC isoforms. (**B**) The predicted BrC binding motif in the regulatory region of Leb3 based on BRC protein from JASPAR (Accession number: MA0012). (**C**) The relative wild-type and mutant (mutant in BrC binding motif) *Leb3* promoter activity after overexpressing three BrC isoforms. (**D**) EMSA analysis of the BrC binding motif of the *Leb3* gene binding to nuclear proteins isolated from the *BmN* cells transfected with pIZ-BrC-Z4 or pIZ-vector. (**E**) The effect of BrC-N isoform on the BrC-mediated activation of AMP promoter activity. The data show mean ± SEM from three independent biological replicates. Significance was calculated using Student’s *t*-test (* *p* < 0.05; ** *p* < 0.01).

Because all three AMP promoters contained abundant potential BrC binding sites, we further examined whether BrC directly regulates their transcription (Figure 4A). The coding sequences of the four BrC splice variants were individually cloned into expression vectors and co-transfected with AMP reporter plasmids into *BmN* cells. The results showed that different BrC isoforms displayed distinct promoter preferences. The *CecE* promoter was specifically activated by BrC-Z4. The *Glv2* promoter was activated by both BrC-Z1 and BrC-Z2. In contrast, the *Leb3* promoter could be strongly activated by BrC-Z1, BrC-Z2, and BrC-Z4 (Figure 5A). To further verify the direct binding of BrC to AMP promoters, we introduced point mutations into the predicted BrC binding motif within the Leb3 regulatory region. The mutant reporter plasmid was then co-transfected with BrC-Z1, BrC-Z2, or BrC-Z4 expression vectors (Figure 5B). Compared with the wild-type promoter, mutation of the BrC binding site significantly reduced 20E/BrC-mediated transcriptional activation (Figure 5C).

Furthermore, EMSA experiments demonstrated that nuclear extracts from BrC-Z4-overexpressing cells formed a specific DNA–protein complex with the BrC binding region of the Leb3 promoter. This interaction was completed by excess unlabeled probes containing the wild-type binding sequence but not by probes containing the mutated sequence (Figure 5D). These results demonstrate that BrC directly binds to cis-regulatory elements in AMP promoters and mediates 20E-induced AMP transcription.

### 3.6. BrC-N Enhances BrC-Mediated Activation of Leb3 Transcription

In this study, we identified a new splice variant BrC-N, which lacks the zinc finger DNA-binding domain. Consistent with the structural analysis, overexpressing *BrC-N* failed to activate any AMP promoter (Figure 5A). This result confirms that loss of the DNA-binding domain eliminates its direct transcriptional activation ability.

Interestingly, although BrC-N lacks transcriptional activation activity, its expression was strongly induced by both 20E and bacterial infection, suggesting a possible non-classical regulatory function. We hypothesized that BrC-N may regulate target gene expression through protein–protein interactions. To test this possibility, BrC-N was co-expressed with BrC-Z1, BrC-Z2, or BrC-Z4, and their combined effects on the Leb3 promoter were examined. Compared with individual expression of BrC-Z isoforms, co-expression with BrC-N further increased Leb3 promoter activity (Figure 5E).

In summary, our results suggest that although BrC-N cannot directly bind DNA due to the absence of the zinc finger domain, its conserved N-terminal BTB domain may mediate heterodimer formation with BrC-Z proteins. Through this interaction, BrC-N may enhance BrC-Z-dependent transcriptional output without altering DNA-binding specificity. This finding reveals a previously unknown regulatory mechanism of BrC isoforms in 20E-mediated immune regulation.

## 4. Discussion

20-hydroxyecdysone (20E) has long been recognized as the major hormone controlling insect molting and metamorphosis [15]. Increasing evidence has shown that 20E also plays an important role in innate immune regulation [16,17,18,19,20,21,22,23,24]. In this study, we used silkworms infected with both Gram-negative bacteria and Gram-positive bacteria as a model to investigate the immune function of 20E. Our results showed that 20E pretreatment significantly reduced bacterial loads after infection and increased the survival rate of silkworms. Further analysis indicated that the protective effect of 20E was mainly achieved by promoting the transcription of antimicrobial peptides (AMPs), which are key effectors of humoral immunity.

At the organismal level, exogenous 20E treatment significantly improved the resistance of silkworms to both Gram-negative and Gram-positive bacterial infections. This result is consistent with previous findings by Sun et al. (2016), who reported that bacterial infection increased endogenous 20E levels in silkworms and enhanced innate immune defense through the EcR receptor pathway [22]. Studies in *Helicoverpa armigera* also showed that 20E treatment induced the expression of pattern recognition receptors and AMPs, leading to increased antibacterial activity in hemolymphs [43,44]. Our study further supports a positive role of 20E in humoral immunity in lepidopteran insects. Importantly, 20E treatment promoted bacterial clearance rather than directly killing bacteria, suggesting that 20E enhances host defense by activating immune responses. This possibility is consistent with previous studies showing that 20E promotes immune cell activity, phagocytosis, and hemocyte differentiation in *Drosophila* [17,45].

The classical regulation model of the 20E signaling pathway involves the activation of downstream early-response transcription factors by the EcR/USP heterodimer, which subsequently controls the temporal expression of target genes. In this study, bioinformatic analysis revealed that BrC-Z binding sites are widely distributed in the promoter regions of most AMP genes and are highly enriched in several genes. These findings suggest that BrC may serve as an important regulatory hub connecting 20E signaling with AMP transcriptional responses. Furthermore, dual-luciferase reporter assays, promoter mutation analysis, and EMSA experiments demonstrated that BmBrC-Z1, Z2, and Z4 can directly recognize and bind to specific cis-regulatory elements in AMP promoters, thereby activating the transcription of antimicrobial peptide genes, including *Leb3*. It should be noted that 20E treatment induced Leb3 reporter activity more strongly than a single *Broad* isoform alone, suggesting that additional 20E-activated transcription factors may cooperate with Broad to drive the full response. These results are consistent with previous studies in *Drosophila*. BR-C and other factors, such as Pannier and Serpent, have been reported as key downstream transcription factors of the 20E pathway and directly regulate AMP gene expression [16,46]. Together, these findings indicate that BR-C is not only a downstream mediator of 20E signaling but also functions as an integration node. Through interactions with different immune regulators, BR-C may coordinate and amplify signals between endocrine regulation and immune responses.

Importantly, this study identified and functionally characterized a novel BrC isoform, BrC-N, which lacks the C-terminal zinc finger domain. Unlike BrC-Z isoforms with DNA-binding activity, BrC-N cannot directly recognize target gene promoters but retains a complete N-terminal BTB/POZ domain. The BTB/POZ domain is a highly conserved protein interaction module that is widely found in transcriptional regulators. It mainly participates in protein dimerization, complex formation, and transcriptional regulation [47]. Therefore, BrC-N may not regulate gene expression through direct DNA binding. Instead, it may interact with other BrC isoforms or regulatory proteins through its BTB domain. Consistent with this, 20E induces all four BrC isoforms, and the newly identified BrC-N enhances the induction capacity of other BrC isoforms, suggesting that isoform interactions may further amplify the transcriptional response. This hypothesis is supported by previous studies. In *Drosophila*, BR-C can physically interact with Relish, a key transcription factor of the IMD pathway, and jointly promote AMP gene expression [46]. In addition, Cheng et al. (2014) identified RACK1 as a BR-C-interacting protein in silkworms [48]. Disruption of the BR-C–RACK1 interaction or deletion of the BTB domain impaired BR-C nuclear localization and reduced its transcriptional activity. These studies indicate that the BTB domain not only determines the dimerization ability of BR-C proteins but also contributes to their subcellular localization and transcriptional regulation.

In summary, we propose that BrC-N may form heterodimers with BrC-Z isoforms through its BTB domain, thereby enhancing BrC-Z-mediated transcriptional activation. This DNA-binding-independent regulatory mechanism provides a new explanation for the functional cooperation among BrC isoforms and highlights the important role of the BTB/POZ domain in integrating insect hormone signaling and immune responses. Overall, this study proposes a new 20E–BrC–AMP regulatory model, in which 20E induces the expression of different BrC isoforms and regulates immune effector genes through the combinatorial control of BrC proteins. This mechanism expands our understanding of the interaction between endocrine regulation and innate immunity in insects and provides new insights into how hormonal signals regulate complex immune networks. Moreover, as the important hub of the hormone and immunity pathway, BrC could be a potential target for pest control.

## Data Availability

The original contributions presented in this study are included in the article and Supplementary Materials. Further inquiries can be directed to the corresponding authors.

## Supplementary Materials

The following supporting information can be downloaded at: https://www.mdpi.com/article/10.3390/insects17101076/s1, Figure S1: Multiple alignment of four silkworm BrC proteins. Protein domains were predicted using the SMART database and are highlighted as follows: BTB domain: red box; C2H2 Zn finger region (ZnF C2H2): black box. Figure S2: Bacterial infection can elevate the 20E concentration. Hemolymph was collected from day-3 fifth-instar larvae at 12 h after bacterial infection to measure 20E concentration. Data are presented as mean ± SEM from three independent biological replicates (*n* = 3 per group). Significance was determined by one-way ANOVA followed by Tukey’s post hoc test for multiple comparisons; * *p* < 0.05. Table S1: Primer list. Table S2: The predicted transcription factor binding site of *AMP* promoters.
