# Supplementary material for: Broad-Complex Mediates 20-Hydroxyecdysone-Induced Antimicrobial Peptide Expression in the Silkworm Bombyx mori
Source: Insects. 2026 Oct 17;17(10):1076. doi: 10.3390/insects17101076 (PMC13705130; doi:10.3390/insects17101076)
Supplement: Supplementary file 1 [file insects-17-01076-s001.zip › insects-4589816-SM .pdf]

Supplemental Materials

Supplemental Figure legend

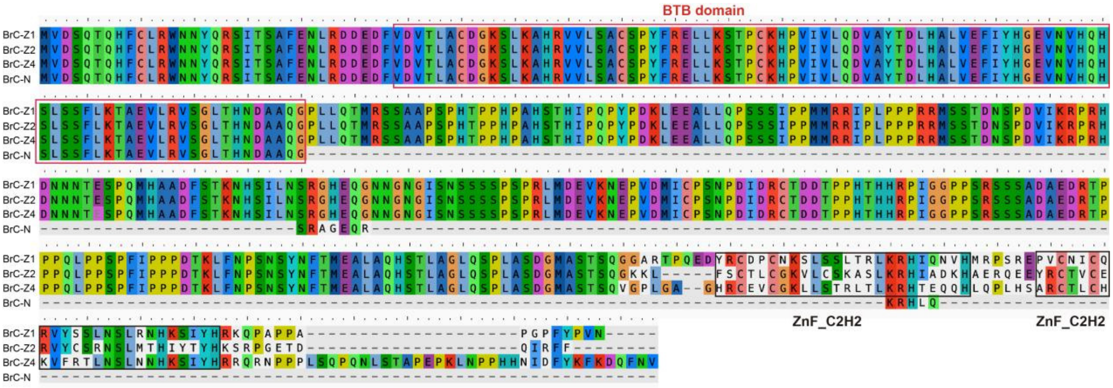

Figure S1. Multiple alignment of four silkworm BrC proteins. Protein domains were predicted using the SMART database and are highlighted as follows: BTB domain: red box; C2H2 Zn finger region (ZnF C2H2): black box.

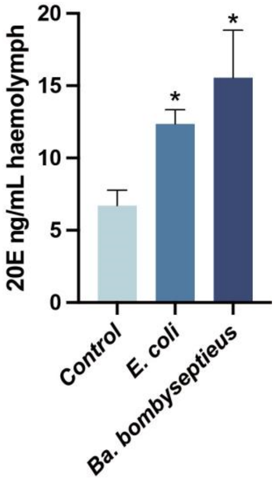

Figure S2. Bacterial infection could elevate the 20E concentration. Hemolymph was collected from day-3 fifth-instar larvae at 12 h after bacterial infection to measure 20E concentration. Data are presented as mean ± SEM from three independent biological replicates (n = 3 per group). Significance was determined by one-way ANOVA followed by Tukey's post hoc test for multiple comparisons; **P* < 0.05.

Supplemental Table S1. Primers list

Usage	Gene names	Primer sequences (5' to 3') Sense-primer	Antisense-primer
RT-PCR	Mor1	TTGTGGCAATGTCTCTGGTGT	ACATCGTTGGCTGTACTGGCG
	CeCB1	ATGAACTTCGCAAAGATCCTAT	TATTTTCCTATGGCTTTAGCCG
	CeCD	ATAGTATTTATCGCCATCAT	TCCTTTGATTCAGAACTGTCG
	CeCE	ATGAATTTCTCAAGAGCTCTGT	TCATTTCCCATGAGCGATGGTC
	LeB3	TTCATCCAGCCGACCTACAG	GGTGACACTGCGAACATACC
	Glv2	ACTCGATCAGCGGGCAATCC	AAGCCTGACCGGTTAGTTTGC
	Glv3	ACGCAGAAGTATACAGGTCT	ACCGGCTTTACCGAATAGTC
	rPL3	CGGTGTTGTTGGATACATTGAG	GCTCATCCTGCCATTTCTTACT
	BrC-Z1	CGCACCCCCCAAGAAGATTA	GTTGACGGGGTAGAAGGGGC
	BrC-Z2	GTAAAAAGTTATTCTCGTGT	AAACCGGATTTGATCCGTC
	BrC-Z4	CCACCGGTGCGAGGTGTGCGGCAA	AGTTTGGGCTCCGGCGCAGT
	BrC-N	ACACAATGACGCTGCCCAGGG	TTTCCGTTGTTCCCCTGCTCGT
Ectopic expression in *BmN* cells	*BrC-Z1*	CGCGGATCCATGGTGGACAGTCAGACG	GCGGCCGCTTACAGATCCTCTTCTGAGATGAG TTTTTGTTCGTTGACGGGGTAGAAGGG
	BrC-Z2	CGCGGATCCATGGTGGACAGTCAGACG	GCGGCCGCTTACAGATCCTCTTCTGAGATGAG TTTTTGTTCAAAAAACCGGATTTGATCCGT
	BrC-Z4	CGCGGATCCATGGTGGACAGTCAGACG	GCGGCCGCTTACAGATCCTCTTCTGAGATGAG TTTTTGTTCGACATTGAATTGGTCTTTGAAT
	BrC-N	CGCGGATCCATGGTGGACAGTCAGACG	GCGGCCGCTTACAGATCCTCTTCTGAGATGAG

			TTTTTGTTCTTGGAGATGCCGTTTCCGTTG
Dual luciferase assay	*CeCE-promoter*	CTAGCTAGCTAGGGACCTGAGCGGTTCTATTGAACA	CCCAAGCTTGGGAATTACAAATGTCCACAC
	Glv2-promoter	CTAGCTAGCTAGGCCGTTGTACTGTAAAAC	CCCAAGCTTGGGCATTTTGAGCTAAAACAA
	Leb3-promoter	CTAGCTAGCTAGACAAAAACCTGAATCTCA	CCCAAGCTTGGGATGTTGATTGTATCGATT
Site mutagenesis	*Leb3-mut-promoter*	AAACATTTGCTTGCACAATT	TTTTCACAGATAGGGACACA
EMSA probes	*Leb3-wt-BrC*	TTTCACAGATAGGTAAACAAAATTTACGAC	GTCGTAAATTTTGTTTACCTATCTGTGAAA
	Leb3-mut-BrC	TTTCACAGATAGGCCCCCAAAATTTACGAC	GTCGTAAATTTTGGGGGCCTATCTGTGAAA

Note: restriction enzyme sites were labeled with single line; Myc tag sequences were labeled with double lines.

Supplemental Table S2. The predicted Transcription factor binding site of *AMPs* promoter

Gene	Matrix	Detailed Matrix Information	position		Strand	Sequence
			From	To		
Glv1	I$E74A.01	E74A early ecdysone-inducible gene in onset of Drosophila metamorphosis	579	593	-	ttatctcGGAAgacg
	I$FTZ.01	Fushi tarazu, involved in body segmentation of the drosophila embryo	338	352	-	tgCTTAatctgccgt
Glv2	I$BRCZ2.01	Broad-Complex Z2 Zinc Finger isoform	302	320	+	ttcgaaaaAATAaaaatta
	I$BRCZ2.01	Broad-Complex Z2 Zinc Finger isoform	530	548	-	agcaaaaaAATAatacaaa
	I$BRCZ3.01	Broad-Complex Z3 Zinc Finger isoform	286	304	-	gaatagaAAACtaaaaagt
	I$BRCZ3.01	Broad-Complex Z3 Zinc Finger isoform	543	561	-	gtccactAAACtaagcaaa
	I$BRCZ3.01	Broad-Complex Z3 Zinc Finger isoform	873	891	-	tttgacaAAACtatacttg
	I$E74A.01	E74A early ecdysone-inducible gene in onset of Drosophila metamorphosis	436	450	+	cttacacGGAAgatt
	I$FTZ.01	Fushi tarazu, involved in body segmentation of the drosophila embryo	270	284	+	acCTTCattgtctaa
	I$FTZ.01	Fushi tarazu, involved in body segmentation of the drosophila embryo	509	523	-	taCTTActtgtatca
Glv3	I$BRCZ1.01	Broad-Complex Z1 Zinc Finger isoform	1196	1214	+	tacttgaaaACAAataatt
	I$BRCZ2.01	Broad-Complex Z2 Zinc Finger isoform	452	470	-	acgtacaaATTAgtaatgc
	I$EIP74EF.01	Ecdysone-induced protein 74EF	232	246	-	ataataaGGAActtc
	I$EIP74EF.01	Ecdysone-induced protein 74EF	563	577	+	tccataaGGAActtt
	I$FTZ.02	Fushi tarazu, involved in body segmentation of the drosophila embryo	760	774	-	tagtTAATtcagaat
	I$FTZ.01	Fushi tarazu, involved in body segmentation of the drosophila embryo	1476	1490	+	atCTTTattattgtt
Glv4	I$BRCZ1.01	Broad-Complex Z1 Zinc Finger isoform	557	575	+	aactcattaACAAaattat
	I$BRCZ1.01	Broad-Complex Z1 Zinc Finger isoform	1400	1418	+	ttattttacACAAaagttt
	I$BRCZ2.01	Broad-Complex Z2 Zinc Finger isoform	118	136	+	aaaaaataAATAataaaaa
	I$BRCZ3.01	Broad-Complex Z3 Zinc Finger isoform	569	587	+	aaattatAAACtacagata
	I$BRCZ3.01	Broad-Complex Z3 Zinc Finger isoform	896	914	-	taacacaAAACtaactttt
	I$BRCZ4.01	Broad-Complex Z4 Zinc Finger isoform	34	52	+	aaatttaaTAAAaataaag
CeCA1	I$BRCZ2.01	Broad-Complex Z2 Zinc Finger isoform	29	47	-	acggcagaAATAggaaggg
	I$BRCZ2.01	Broad-Complex Z2 Zinc Finger isoform	215	233	+	aagcaataAATAaaaaaag
	I$BRCZ4.01	Broad-Complex Z4 Zinc Finger isoform	990	1008	+	ctatattaTAAAtacaatc
	I$EIP74EF.01	Ecdysone-induced protein 74EF	28	42	-	agaaataGGAAgggt
	I$FTZ.01	Fushi tarazu, involved in body segmentation of the drosophila embryo	826	840	-	caCTTAatgccaagt
	I$FTZ.01	Fushi tarazu, involved in body segmentation of the drosophila embryo	1140	1154	+	aaCTTAaatgtgcta
CeCA2	I$BRCZ1.01	Broad-Complex Z1 Zinc Finger isoform	630	648	-	tacctattgACAAaagctc

	I$BRCZ1.01	Broad-Complex Z1 Zinc Finger isoform	1045	1063	-	aaataatagTCAAtaaaat
	I$BRCZ4.01	Broad-Complex Z4 Zinc Finger isoform	973	991	+	ctatattaTAAAtacaatc
	I$E74A.01	E74A early ecdysone-inducible gene in onset of Drosophila metamorphosis	435	449	+	taaatccGGAAcgcg
	I$EIP74EF.01	Ecdysone-induced protein 74EF	219	233	-	atgtaaaGGAAgatt
	I$FTZ.01	Fushi tarazu, involved in body segmentation of the drosophila embryo	830	844	-	caCTTAatgccaagt
	I$FTZ.01	Fushi tarazu, involved in body segmentation of the drosophila embryo	1092	1106	-	tcCTTTattggactt
CeCB1	I$BRCZ1.01	Broad-Complex Z1 Zinc Finger isoform	1322	1340	-	aggttaaacACAAacaaca
	I$BRCZ1.01	Broad-Complex Z1 Zinc Finger isoform	1388	1406	-	tttatatatACAAattcgc
	I$BRCZ2.01	Broad-Complex Z2 Zinc Finger isoform	674	692	-	gcatataaAATAgtgacag
	I$BRCZ2.01	Broad-Complex Z2 Zinc Finger isoform	1101	1119	+	caataaaaAATAaaaataa
	I$BRCZ3.01	Broad-Complex Z3 Zinc Finger isoform	272	290	-	tgacaatAAACaaatgata
	I$BRCZ4.01	Broad-Complex Z4 Zinc Finger isoform	274	292	-	tttgacaaTAAAcaaatga
	I$BRCZ4.01	Broad-Complex Z4 Zinc Finger isoform	344	362	-	ggtattaaTAAAgacaaac
	I$BRCZ4.01	Broad-Complex Z4 Zinc Finger isoform	692	710	-	atattttaTAAAaaaaaag
	I$BRCZ4.01	Broad-Complex Z4 Zinc Finger isoform	1103	1121	+	ataaaaaaTAAAaataaaa
	I$E74A.01	E74A early ecdysone-inducible gene in onset of Drosophila metamorphosis	1222	1236	-	gtcaaccGGAAaatg
CeCB2	I$BRCZ1.01	Broad-Complex Z1 Zinc Finger isoform	1391	1409	-	tttatatatACAAattcgc
	I$BRCZ2.01	Broad-Complex Z2 Zinc Finger isoform	587	605	+	ccaaaaaaAATAacaataa
	I$BRCZ2.01	Broad-Complex Z2 Zinc Finger isoform	1100	1118	+	caataaaaAATAaaaataa
	I$BRCZ4.01	Broad-Complex Z4 Zinc Finger isoform	1102	1120	+	ataaaaaaTAAAaataaaa
	I$BRCZ4.01	Broad-Complex Z4 Zinc Finger isoform	1398	1416	+	tgtatataTAAAgaaaaca
	I$E74A.01	E74A early ecdysone-inducible gene in onset of Drosophila metamorphosis	1221	1235	-	gtcaaccGGAAaatg
	I$FTZ.01	Fushi tarazu, involved in body segmentation of the drosophila embryo	595	609	-	caCTTTattgttatt
	I$FTZ.01	Fushi tarazu, involved in body segmentation of the drosophila embryo	1087	1101	-	tgCTTAgttgggtgg
CeCD	I$BRCZ1.01	Broad-Complex Z1 Zinc Finger isoform	13	31	-	ttattatttACAAaaaatg
	I$E74A.01	E74A early ecdysone-inducible gene in onset of Drosophila metamorphosis	138	152	+	gtcactcGGAAataa
	I$FTZ.01	Fushi tarazu, involved in body segmentation of the drosophila embryo	1255	1269	-	atCTTAtttgtagtt
CeCE	I$BRCZ2.01	Broad-Complex Z2 Zinc Finger isoform	1332	1350	+	acaaaacaAATAaaaactt
	I$BRCZ2.01	Broad-Complex Z2 Zinc Finger isoform	2375	2393	-	aaatgcaaATTAgaaaaaa
	I$BRCZ4.01	Broad-Complex Z4 Zinc Finger isoform	1247	1265	+	aactaaaaGAAAcaaaaca
	I$E74A.01	E74A early ecdysone-inducible gene in onset of Drosophila metamorphosis	1234	1248	+	acaaaacGGAAataa
LEB3	I$BRCZ4.01	Broad-Complex Z4 Zinc Finger isoform	220	238	-	cagataggTAAAcaaaatt
